# Primum Non Nocere: A Case Report of Iatrogenic Fracture of the Mandibular Angle During Excision of an Impacted Third Molar

**DOI:** 10.7759/cureus.27672

**Published:** 2022-08-04

**Authors:** Pooja Agrawal, Anendd Jadhav, Nitin D Bhola

**Affiliations:** 1 Oral and Maxillofacial Surgery, Sharad Pawar Dental College and Hospital, Datta Meghe Institute of Medical Sciences, Wardha, IND

**Keywords:** primum non nocere, third molar complications, third molar extraction, mandibular angle fracture, iatrogenic fracture

## Abstract

Third molar extractions are one of the most commonly performed dental procedures. It is associated with numerous complications, of which mandibular angle fracture is a rare but distressing complication. These can occur as intraoperative and postoperative (late) events. Iatrogenic fractures involving the angle of the mandible represent a unique challenge for management owing to their complex biomechanics and various anatomical factors. Intraoperative fractures occur due to various reasons, which include the position of the tooth, depth of impaction, extent of odontectomy performed, and injudicious use of dental elevators. This exhibited report describes a case of iatrogenic mandibular angle fracture (IFM) during excision of an impacted third molar in a 30-year-old female. Additionally, it discusses the various reasons and preventive strategies to avoid such complications.

## Introduction

The face is one of the most often injured areas of the body. It accounts for 23%-97% of facial fractures [1]. Surgical excision of the impacted third molar is the most routinely performed procedure in daily clinical practice [2]. It is accompanied by certain intraoperative and postoperative complications such as edema, pain, trismus, nerve injury, and tooth displacement [1]. The associated mandibular fracture is a dreaded and exceedingly infrequent complication having multifactorial etiology and prevalence ranging between 0.0034% and 0.0075% [3].

The mandibular angle is one of the inherently vulnerable regions prone to fracture owing to complex biomechanics and anatomical characteristics. It is estimated that the existence of an impacted third molar decreases the cross-sectional area of bone and leads to a twofold to fourfold increase in fractures of the mandibular angle [4]. Mandibular angle fractures pose a unique challenge for surgeons because the masseter and medial pterygoid muscles are attached to the angle. These can cause displacement of bone fragments after a fracture. Fracture of the mandibular angle after exodontia is rare and usually underreported [5]. Thus, the main aim of this report is to exhibit a case of iatrogenic mandibular angle fracture (IFM) while excising the impacted third molar and discuss the various preventive and management strategies concerning the same.

## Case presentation

A systemically healthy 30-year-old countryside female visited our outpatient section of the department with a chief complaint of pain and swelling over the lower right side of her face with an inability to open her mouth and chew for four days. The patient had a history of a traumatic surgical procedure to extract her lower right impacted third molar under local anesthesia elsewhere.

On examination, the face was grossly asymmetrical due to diffuse swelling present over the lower right region of the face extending anteroposteriorly distal to the corner of the lip to the posterior border of the ramus of the mandible and supero-inferiorly from the level of the corner of the mouth to 2 cm beyond the lower border of the mandible. The overlying skin was the same color as the adjacent skin, afebrile on touch, and tender on palpation. Detailed palpation could not be performed as the swelling was tender, and restricted mouth opening did not permit intraoral examination. The occlusion was bilaterally stable, and at rest, no abnormality was detected.

**Figure 1:**
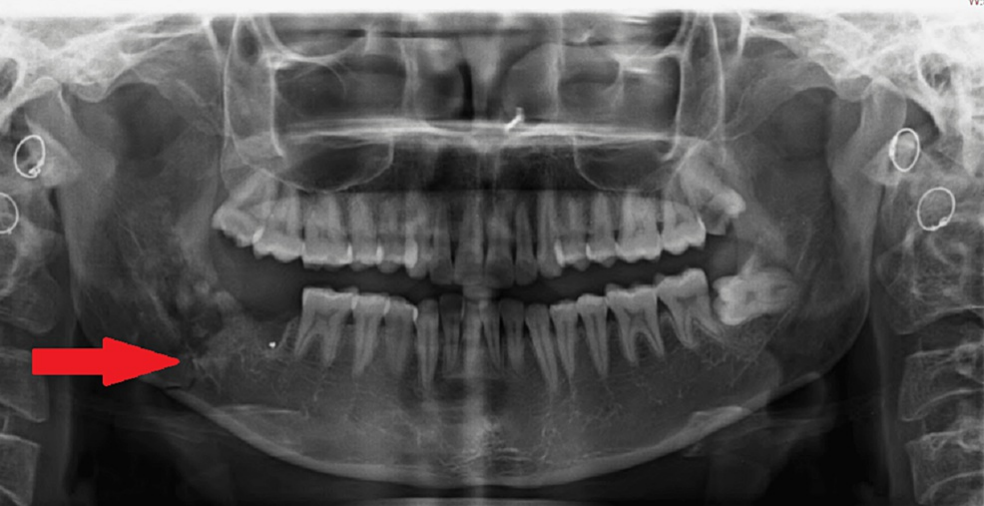
Orthopantomogram showing oblique radiolucent line extending from the extraction socket of the right third molar fracturing the right angle of the mandible (arrow).

**Figure 2:**
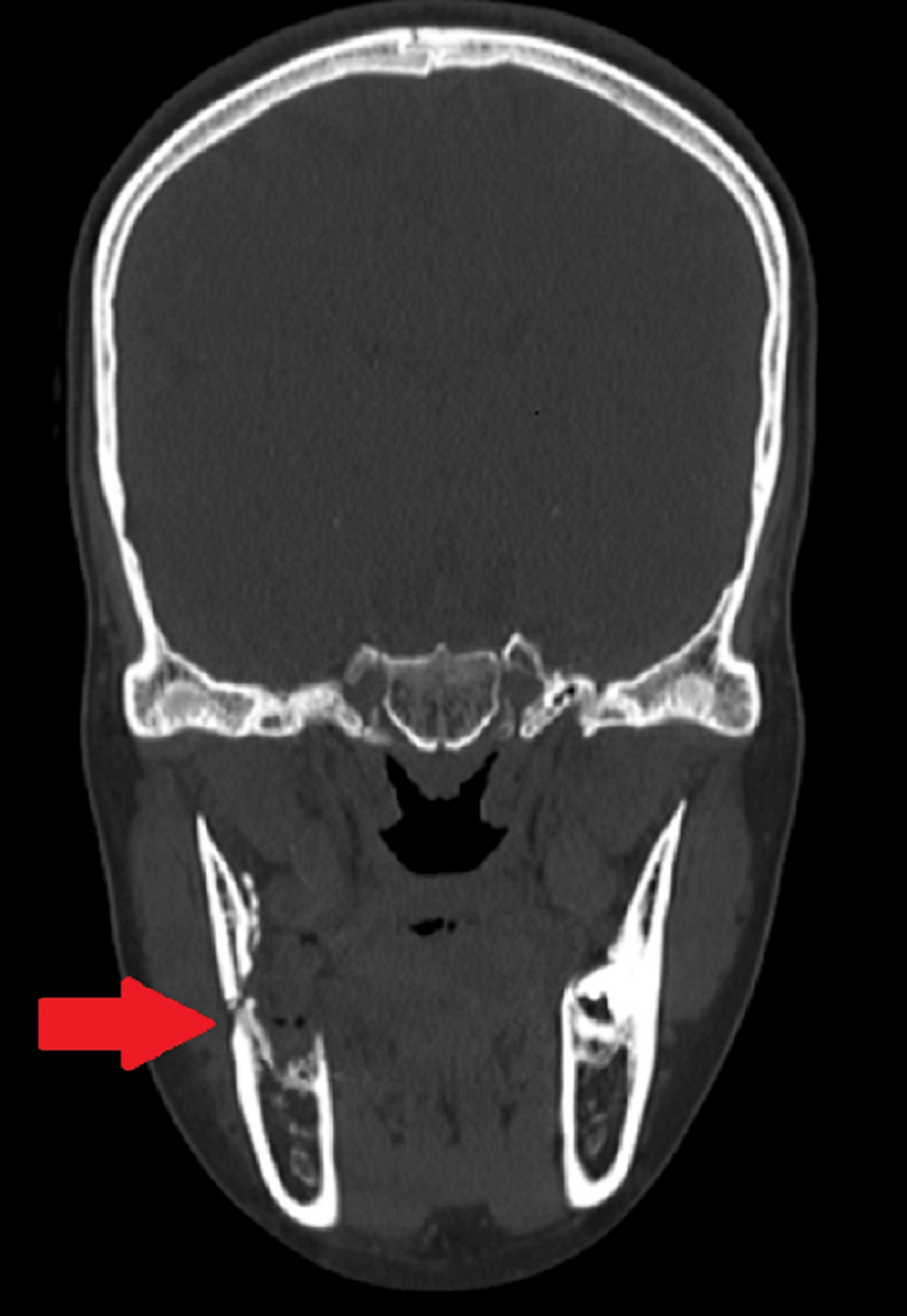
Coronal cut showing right mandibular angle fracture (arrow).

An orthopantomogram (OPG) was done, which demonstrated extracted second and third molar socket through which a thin radiolucent line was seen running obliquely from the alveolar crest of the extraction site to the angle of the mandible, indicating a fracture (Figure 1). A computed tomography (CT) scan (Figure 2) was advised, which demonstrated a tooth socket with gross destruction of the lingual cortical plate and a breach in the continuity of bone through the socket, indicating a mandibular angle fracture.

**Figure 3:**
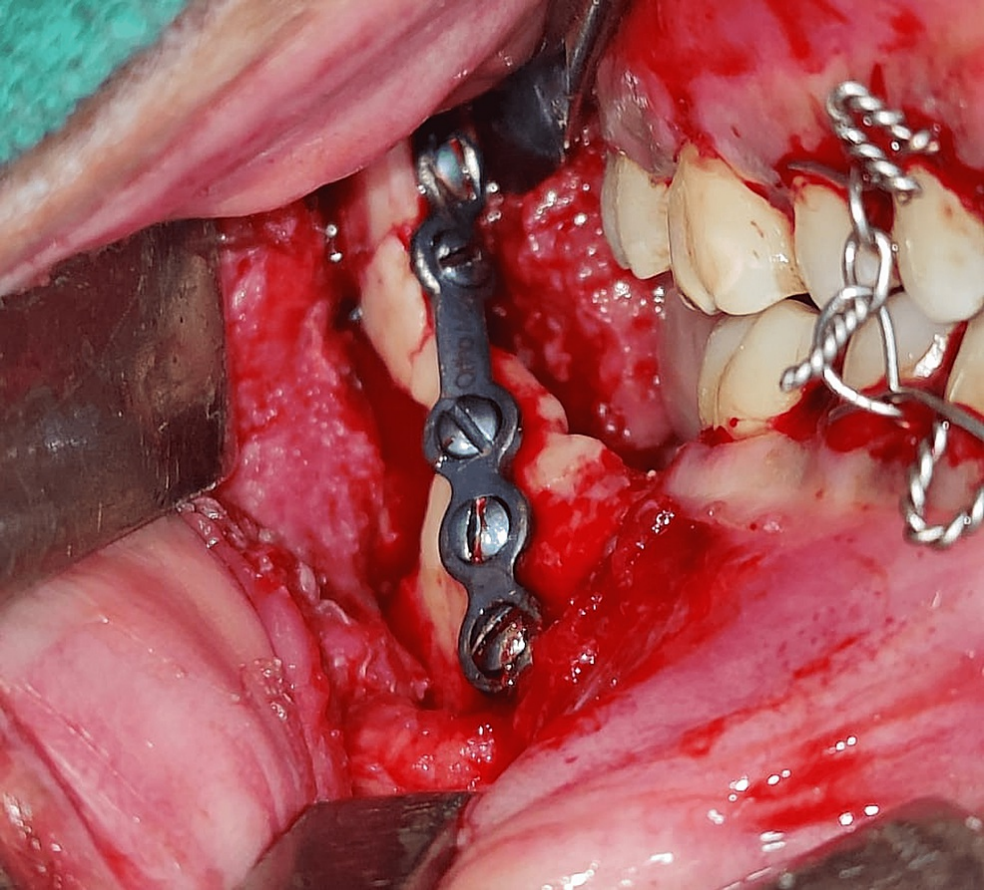
Plating of the superior border via the transoral approach.

**Figure 4:**
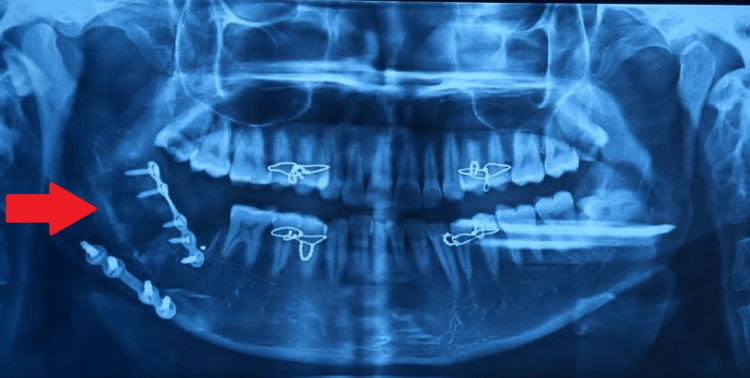
Postoperative orthopantomogram showing fixation of the superior border and lateral border with miniplates and screws (arrow).

Upon resolution of the edema, the fracture was treated with open reduction and two-point fixation, one superior border plating using miniplates via a transoral approach, and lateral border plating using a trans-buccal approach with titanium plates and screws (Figure 3). Postoperatively, antibiotics and analgesics were prescribed, a check OPG was done (Figure 4), and the patient was discharged on the fifth day; the recovery was uneventful.

## Discussion

Intraoperative iatrogenic mandibular angle fractures occurring as a sequela of dental extraction are a rare but serious complication arising as an abrupt or late event. Late mandibular fractures tend to occur more frequently compared to immediate fractures [6]. The etiology is thought to be multifactorial, including age, gender, the position of the tooth, the volume of the tooth, bone present, the depth of impaction, the extent of osteotomy performed as well as the surgical technique employed, and certain systemic conditions that influence bone metabolism [7]. Intraoperative mandibular angle fractures with late reporting are often challenging as a detailed clinical examination is difficult to perform due to pain, edema, and trismus. Therefore, a clinician is left at the discretion of radiological examination for diagnosis. However, conventional radiography often lacks accuracy [7]. CT scan is considered a gold standard in such a scenario. In the present case, the diagnosis was established using an orthopantomogram and confirmed using a CT scan.

Intraoperative jaw fractures are a direct result of an application of excessive force and injudicious use of instrumentation. There seems to be a general agreement among authors of the reported cases regarding the age of occurrence and gender of subjects sustaining iatrogenic mandibular fractures. Concerning age and gender, reduction of the elasticity of bone, narrowing of the periodontal ligament space, ankylosis of tooth, and greater masticatory force can be considered plausible attributing factors [8]. In the present case, a contradictory trend is observed, in which a female of 30 years sustained the injury.

Before dental extractions, the total volume of the mandible that is being occupied by the impacted tooth is also an essential element in assessing the risk. To avoid IFM, the tooth/jawbone ratio should be evaluated using an orthopantomogram, cone beam computed tomography (CBCT), or CT scan. Wagner et al. reported this ratio to be 62% [9], whereas Iizuka et al. reported that the ratio varied from 44% to 84% [10]. The depth of tooth impaction is an essential factor to be considered. A partially impacted tooth reduces a small cross-sectional area of bone at the upper border of the mandible, which leads to fragility at the angle region. Complete bony impactions are associated with greater difficulty in the removal and extensive osteotomy, translating into the weakening of mandibular bone and fracture. In the present case, the influence of the depth of the impacted tooth on mandibular fractures was not known.

Another reason for IFM could be the presence of former bony pathologies such as cysts and tumors, recurrent pericoronitis, and periodontal disease, all commonly occurring lesions in the mandibular angle region. Due to the reasons mentioned above, the mandible becomes weakened, which further predisposes to fracture. An incorrect surgical technique or injudicious use of elevators or forceps is also a consideration for the incidence of iatrogenic fracture of the mandible, which should be avoided by taking necessary precautions. The risk of fracture is even higher among more inexperienced surgeons.

The management strategies for mandibular angle fractures are diverse and range from no treatment, soft diet, and intermaxillary fixation to open reduction and internal fixation. Treatment options are dictated by the characteristics of a fracture and the surgeon’s preference [6]. In the present case, after explaining all the treatment options, the patient opted for open reduction over intermaxillary fixation. The patient was treated with two-point fixation with miniplates, one at the upper border placed transorally and the other laterally over the lower border of the mandible via a trans-buccal approach. The postoperative recovery was uneventful, and the patient had a functionally stable occlusion at the conclusion of six months follow-up.

## Conclusions

IFM is a rare condition and can be easily avoided. Besides various causes, the incidence of IFM is relatively low. A detailed preoperative evaluation of the impacted teeth and bone adjacent to the sectioning of the tooth and minimal removal of bone during trans-alveolar extraction are a few techniques that can be employed to avoid IFM. The presence of a third molar increases the incidence of mandibular angle fracture. Although IFM is rare, whenever it occurs is very much distressing to the patients. Thorough knowledge of anatomy, judicious use of elevators and forceps, and sectioning of the tooth as and when required should be implemented practically to avoid IFM.

## References

[1] Cutilli T, Bourelaki T, Scarsella S, Fabio DD, Pontecorvi E, Cargini P, Junquera L (2013). Pathological (late) fractures of the mandibular angle after lower third molar removal: a case series. J Med Case Rep.

[2] Ramkumar Ceyar KA, Thulasidoss GP, Raja Sethupathy Cheeman S, Sagadevan S, Panneerselvam E, Krishna Kumar Raja VB (2020). Effectiveness of knotless suture as a wound closure agent for impacted third molar - a split mouth randomized controlled clinical trial. J Craniomaxillofac Surg.

[3] Bodner L, Brennan PA, McLeod NM (2011). Characteristics of iatrogenic mandibular fractures associated with tooth removal: review and analysis of 189 cases. Br J Oral Maxillofac Surg.

[4] Cankaya AB, Erdem MA, Cakarer S, Cifter M, Oral CK (2011). Iatrogenic mandibular fracture associated with third molar removal. Int J Med Sci.

[5] Adeyemo WL, Ogunlewe MO, Ladeinde AL, Hassan OO, Taiwo OA (2010). A comparative study of surgical morbidity associated with mandibular third-molar surgery in young and aging populations. J Contemp Dent Pract.

[6] Ethunandan M, Shanahan D, Patel M (2012). Iatrogenic mandibular fractures following removal of impacted third molars: an analysis of 130 cases. Br Dent J.

[7] Chrcanovic BR, Abreu MH, Freire-Maia B, Souza LN (2012). 1,454 mandibular fractures: a 3-year study in a hospital in Belo Horizonte, Brazil. J Craniomaxillofac Surg.

[8] Joshi A, Goel M, Thorat A (2016). Identifying the risk factors causing iatrogenic mandibular fractures associated with exodontia: a systemic meta-analysis of 200 cases from 1953 to 2015. Oral Maxillofac Surg.

[9] Wagner KW, Otten JE, Schoen R, Schmelzeisen R (2005). Pathological mandibular fractures following third molar removal. Int J Oral Maxillofac Surg.

[10] Iizuka T, Tanner S, Berthold H (1997). Mandibular fractures following third molar extraction. A retrospective clinical and radiological study. Int J Oral Maxillofac Surg.

